# Systematic review and literature appraisal on methodology of conducting and reporting critical-care echocardiography studies: a report from the European Society of Intensive Care Medicine PRICES expert panel

**DOI:** 10.1186/s13613-020-00662-y

**Published:** 2020-04-25

**Authors:** S. Huang, F. Sanfilippo, A. Herpain, M. Balik, M. Chew, F. Clau-Terré, C. Corredor, D. De Backer, N. Fletcher, G. Geri, A. Mekontso-Dessap, A. McLean, A. Morelli, S. Orde, T. Petrinic, M. Slama, I. C. C. van der Horst, P. Vignon, P. Mayo, A. Vieillard-Baron

**Affiliations:** 1 Intensive Care Unit, Nepean Hospital, The University of Sydney, Sydney, Australia; 2 Department of Anesthesia and Intensive Care, Policlinico-Vittorio Emanuele University Hospital, Catania, Italy; 3 Department of Intensive Care, Erasme University Hospital, Univeristé Libre de Bruxelles, Brussels, Belgium; 4 Department of Anaesthesiology and Intensive Care, 1st Faculty of Medicine, Charles University and General University Hospital, Prague, Czech Republic; 5 Department of Anaesthesiology and Intensive Care, Medical and Health Sciences, Linköping University, Linköping, Sweden; 6 Department of Anaesthesiology and Critical Care Medicine, Vall d’Hebron University Hospital, Barcelona, Spain; 7 Department of Perioperative Medicine, Bart’s Heart Centre St. Bartholomew’s Hospital, W. Smithfield, London, UK; 8 CHIREC Hospitals, Université Libre de Bruxelles, Brussels, Belgium; 9 Cardiothoracic Critical Care, St Georges Hospital, St Georges University of London, London, UK; 10 Intensive Care Medicine Unit, Assistance Publique-Hôpitaux de Paris, University Hospital Ambroise Paré, 92100 Boulogne-Billancourt, France; 11 INSERM, UMR-1018, CESP, Team Kidney and Heart, University of Versailles Saint-Quentin en Yvelines, Villejuif, France; 12 Service de réanimation médicale, Hôpital Henri Mondor, Assistance Publique-Hôpitaux de Paris, 51 Avenue du Maréchal de Lattre de Tassigny, 94000 Créteil, France; 13 Department of Cardiovascular, Respiratory, Nephrological, Anesthesiological and Geriatric Sciences, University of Rome, “La Sapienza,” Policlinico Umberto Primo, Viale del Policlinico, Rome, Italy; 14 Bodleian Health Care Libraries, University of Oxford, Oxford, UK; 15 Medical Intensive Care Unit, Amiens University Hospital, Amiens, France; 16 Department of Intensive Care, Maastricht University Medical Centre+, University Maastricht, Maastricht, The Netherlands; 17 Medical-Surgical Intensive Care Unit, Limoges University Hospital, Inserm CIC 1435, Limoges, France; 18 Division of Pulmonary, Critical Care and Sleep Medicine, Northwell Health LIJ/NSUH Medical Center, Zucker School of Medicine, Hofstra/Northwell, Hempstead, NY USA

**Keywords:** Guidelines, Recommendations, Intensive care, Left ventricle, Right ventricle, Fluid management

## Abstract

**Background:**

The echocardiography working group of the European Society of Intensive Care Medicine recognized the need to provide structured guidance for future CCE research methodology and reporting based on a systematic appraisal of the current literature. Here is reported this systematic appraisal.

**Methods:**

We conducted a systematic review, registered on the Prospero database. A total of 43 items of common interest to all echocardiography studies were initially listed by the experts, and other “topic-specific” items were separated into five main categories of interest (left ventricular systolic function, LVSF *n* = 15, right ventricular function, RVF *n* = 18, left ventricular diastolic function, LVDF *n* = 15, fluid management, FM *n* = 7, and advanced echocardiography techniques, AET *n* = 17). We evaluated the percentage of items reported per study and the fraction of studies reporting a single item.

**Results:**

From January 2000 till December 2017 a total of 209 articles were included after systematic search and screening, 97 for LVSF, 48 for RVF, 51 for LVDF, 36 for FM and 24 for AET. Shock and ARDS were relatively common among LVSF articles (both around 15%) while ARDS comprised 25% of RVF articles. Transthoracic echocardiography was the main echocardiography mode, in 87% of the articles for AET topic, followed by 81% for FM, 78% for LVDF, 70% for LVSF and 63% for RVF. The percentage of items per study as well as the fraction of study reporting an item was low or very low, except for FM. As an illustration, the left ventricular size was only reported by 56% of studies in the LVSF topic, and half studies assessing RVF reported data on pulmonary artery systolic pressure.

**Conclusion:**

This analysis confirmed sub-optimal reporting of several items listed by an expert panel. The analysis will help the experts in the development of guidelines for CCE study design and reporting.

## Background

There is growing use of basic and advanced critical care echocardiography (CCE) as a diagnostic and sequential monitoring tool for decision-making by intensive care physicians. The use of CCE has been defined as echocardiography performed in critically ill patients by intensivists who also interpret the scan results [1], although several CCE studies have involved cardiologists or sonographers. This has been an area of rapid growth over the last decade with consequent demand for training and accreditation processes, in addition to supporting evidence in the field [2, 3].

The Echocardiography Working Group of the Cardiovascular Dynamics section of the European Society of Intensive Care Medicine (ESICM) recognizes that with a growing CCE literature and huge heterogeneity in studies identified by several systematic reviews and meta-analyses of CCE [4–10], there is a need to provide structured guidance for future CCE research methodology, reporting, and interpretation. The aim is to improve CCE research data reporting for future research, to ultimately support clinical decision-making in the monitoring, diagnosis and treatment of critically ill patients.

The Echocardiography Working Group decided to perform first a comprehensive critical appraisal of the available CCE literature to describe current reporting in order to provide evidence for the ultimate aim of PRICES (*Preferred Reporting Items for Critical*-*care Echocardiography Studies)* recommendations. Here we report the results of the systematic review describing the frequency of reporting of items of possible importance for CCE research.

## Methods

### Assembly of expert panel

The PRICES project was initiated by the Echocardiography Working Group of the ESICM. A total of 19 physicians with recognized expertise in the field of CCE were involved from different parts of the World (Europe *n* = 15, Oceania *n* = 3, North America *n* = 1). The first internal discussion regarding the PRICES project started in Vienna (September 25th and 26th, 2017). The authors requested and obtained endorsement by the ESICM. After extensive electronic correspondence, the experts’ group was first assembled in Brussels (March 17th, 2018) where they agreed on:

- the importance of supporting PRICES recommendations with a systematic review on the available research that includes CCE data. This decision was made with the aim of providing a basis for a precise and critical appraisal of the utility of the reported information in current CCE literature according to different domains (i.e. design, methodology, statistics, results reporting, etc.);
- the need to split CCE literature according to specific areas (or “topics”) of interest in CCE research: (1) left ventricular systolic function (LVSF); (2) right ventricular function (RVF); (3) left ventricular diastolic function (LVDF); (4) fluid management (FM), and (5) advanced echocardiography techniques (AET, including speckle tracking and/or 3-D echocardiography studies only);
- the necessity to preventively establish a list of items that should be evaluated during the appraisal of the findings of the systematic search (see “Items and data extraction”).
- the fact that the PRICES did not aim to create unreasonable standards of reporting CCE research which may bias against the publication of future important studies, but to give to the researchers a large amount of information helping them in designing, conducting and reporting their studies.

### Systematic review

#### Literature search

The protocol of the systematic review was registered on PROSPERO database (CRD42018094450) on 1st May 2018. Literature searches using Medline and Embase were made by SH (systematic review coordinator) and TP (professional librarian) in May 2018 and performed separately for each topic/area with tailored search strategies (see Additional file 1). The inclusion period was from 1st January 2000 to 31st December 2017. This period was arbitrarily decided to produce an acceptable workload and because a large increase in the number of CCE publications started since 2000 [3].

#### Screening and studies appraisal

Screenings were performed separately by experts for each topic under the oversight of a designated team leader. Two experts screened each abstract retrieved from the search, and those satisfying all the following criteria were included: (a) critical care population, (b) adult population, (c) reporting echocardiography data in the study, (d) clinical study, (e) English language, and (f) research articles with original data. A third expert was involved to resolve cases of disagreement. We excluded studies where outcome from cardiac surgical conditions and techniques was the primary aim, and where patients were supported by extracorporeal membrane oxygenation or ventricular assist devices. The full-text articles of included abstracts were downloaded and were appraised in detail by two experts to ensure inclusion suitability. Risk of bias assessment was beyond the scope of this appraisal and thus not performed.

#### Items and data extraction

Each included article was searched for a list of pre-determined echocardiographic information (“preferred items” or simply “items”), the absence of which was deemed to potentially introduce bias in measurement, misinterpretation or non-reproducibility of the study results. Such items were proposed during the first expert assembly and classified into “common” ones (study characteristics; patient characteristics; echocardiography information and purpose; clinical information during echocardiography procedure; measurement reliability; statistical analysis) and “topic-specific” (Table 1).

**Table 1:** Lists of various domains and preferred items

	Domains and items
Common to all topics	*Study information (n *= *3)*
	Study type, study design, sample size
	*Patients characteristics (n *= *12)*
	Context
	Age, gender, height and weight (or BMI)
	History of hypertension, HFpEF, HFrEF, ischemic heart disease, atrial fibrillation, COPD, chronic renal failure, presence of pacemaker
	*Echocardiography information (n *= *6)*
	Type of echocardiography; were data collected at end-expiration? Number of beats for data averaging? Was airway pressure trace displayed on screen?
	Vendor of ultrasound machine and software version
	*Clinical information at the time of echocardiography (n *= *10)*
	Mode of ventilation; if mechanically ventilated tidal volume, plateau pressure and positive end-expiratory pressure
	Cardiac rhythm, heart rate, blood pressure; inotropes, vasopressors and their doses
	*Measurement reliability (n *= *8)*
	Feasibility; intra-observer and inter-observer variability; was observer blinded to treatment?
	Echocardiographer professional training and experience in echocardiography
	Reviewer’s professional training and experience in echocardiography
	*Statistics reporting (n *= *4)*
	Was sample size and power calculation provided? Was analysis blinded? Were confounders addressed? Was internal validation provided?
Topic-specific items	*LV systolic function (n *= *15)*
	LV size, LV ejection fraction, LV fractional area change, Tissue Doppler *S*ʹ velocity, MAPSE, LV d*P*/d*t*, LV Tei index, LV strain or strain rate, regional wall motion score
	Cardiac output, stroke volume, presence of heart valve disease; patent foramen ovale; pericardial effusion, tamponade
	*RV function (n *= *18)*
	*RV end*-*diastolic* diameter; RV end-diastolic area; RV-to-LV end-diastolic area ratio; TAPSE; RV fractional area change; tissue Doppler *S*ʹ velocity; RV Tei index; RV strain or strain rate; subjective rating of RV function; PAPs or TR peak velocity; PAAT
	Patent foramen ovale; pericardial effusion; tamponade; RV wall thickness; paradoxical septal motion; IAS bowing; IVC diameter
	*LV diastolic function (n *= *15)*
	*E/A* ratio; tissue Doppler *E*ʹ velocity; *E/E*ʹ ratio; PAPs or TR peak velocity; mitral *E* propagation velocity; mitral *E* deceleration time; pulmonary venous flow; left atrial size
	Systolic, diastolic and mean blood pressure; chronic medications; criteria used for grading diastolic function; guidelines or reference for criteria cited; technical details of measurements
	*Fluid management (n *= *7)*
	Parameter used to predict FR, echocardiographic parameter to assess FR-to-volume challenge or passive leg raising
	Was fluid responsiveness defined? Were technical details of measurements provided? Was reference (“gold”) standard for comparison stated? Was description of the reference standard provided? Was echocardiography used as reference standard?
	*Advanced echocardiography techniques (n *= *17)*
	Types of strain used in LV study; strain or strain rate used in LV study; myocardial layer analysed for LV strain study; RV longitudinal strain, RV longitudinal strain rate; number of cycles used in analysis; start time in cardiac cycle used in analysis, frame rate; number of planes used in analysis; method of image exclusion, method of segments exclusion; details of image optimization method; drift correction used
	Number of beats used in 3-D analysis; frame or volume rate used in 3-D analysis; timing of respiratory cycle in 3-D analysis; reference method in 3-D analysis

Items are divided in common to all critical care echocardiography studies and those of particular interest in a specific topic

*BMI* body mass index, *COPD* chronic obstructive pulmonary disease, *FR* fluid responsiveness, *HFpEF* heart failure with preserved ejection fraction, *HFrEF* heart failure with reduced ejection fraction, *IAS* inter-atrial septum, *IVC* inferior vena cava, *LV* left ventricle, *MAPSE* mitral annulus plan systolic excursion, *PAPs* pulmonary artery systolic pressure, *PAAT* pulmonary artery acceleration time, *RV* right ventricle, *TAPSE* tricuspid annular plan systolic excursion, *TR* tricuspid regurgitation

Most items were categorical and related to whether the items had been reported or not, or in some cases how certain information was collated. Double-data entry method (two different experts blinded to each other) was used for data extraction via a web-based database (REDCap hosted at University of Sydney—https://redcap.sydney.edu.au). Any discrepancy was resolved by a third expert of the same group (“adjudicator”), or eventually referring to a “grand adjudicator” for a final decision. The quality of data extraction was validated by an independent expert methodologist (GG). Briefly, a total of 20 articles were selected randomly (proportionally to the total amount for each topic) and data extracted was compared to those obtained by the experts. A total of 11 discrepancies were found and, considering an average of ~ 60 items per study, the “error” rate was far below 1% per study.

### Data analysis

Data analysis was conducted separately for each topic of CCE interest. From the beginning it was clear that each item did not carry the same importance in different areas of CCE interest. The potential importance of each item and recommendation for its reporting will be the object of the PRICES recommendation paper and are not discussed here since this is a systematic descriptive non-clinical review. In the present study, data on item reporting appraisal are summarized as percentage of items reported per study (PIPS) and as fraction of studies reporting an item (FSi).

PIPS was calculated as a percentage obtained from the sum of items reported in a study divided by the total number of items:

$${\text{PIPS}} = \frac{{{\text{number }}\;{\text{of}}\;{\text{items}}\; {\text{reported}}\; {\text{in}}\; {\text{a}}\; {\text{study}}}}{{{\text{total }}\;{\text{number}}\;{\text{of}}\;{\text{items}} }} \times 100\% .$$

A low PIPS score means the study failed to report a substantial number of items.

FSi was calculated as the total number of studies reporting a particular item divided by the total number of studies:

$${\text{FSi}} = \frac{{{\text{number}}\;{\text{of}}\;{\text{studies}}\; {\text{reporting}}\; {\text{an}}\; {\text{item}}}}{{{\text{Total}}\; {\text{number}}\; {\text{of}}\;{\text{studies }}\;{\text{included}}}}.$$

FSi can be viewed as the “popularity” of an item—the higher FSi means the more studies reported it. The FSi was calculated for all the items.

## Results

**Fig. 1:**
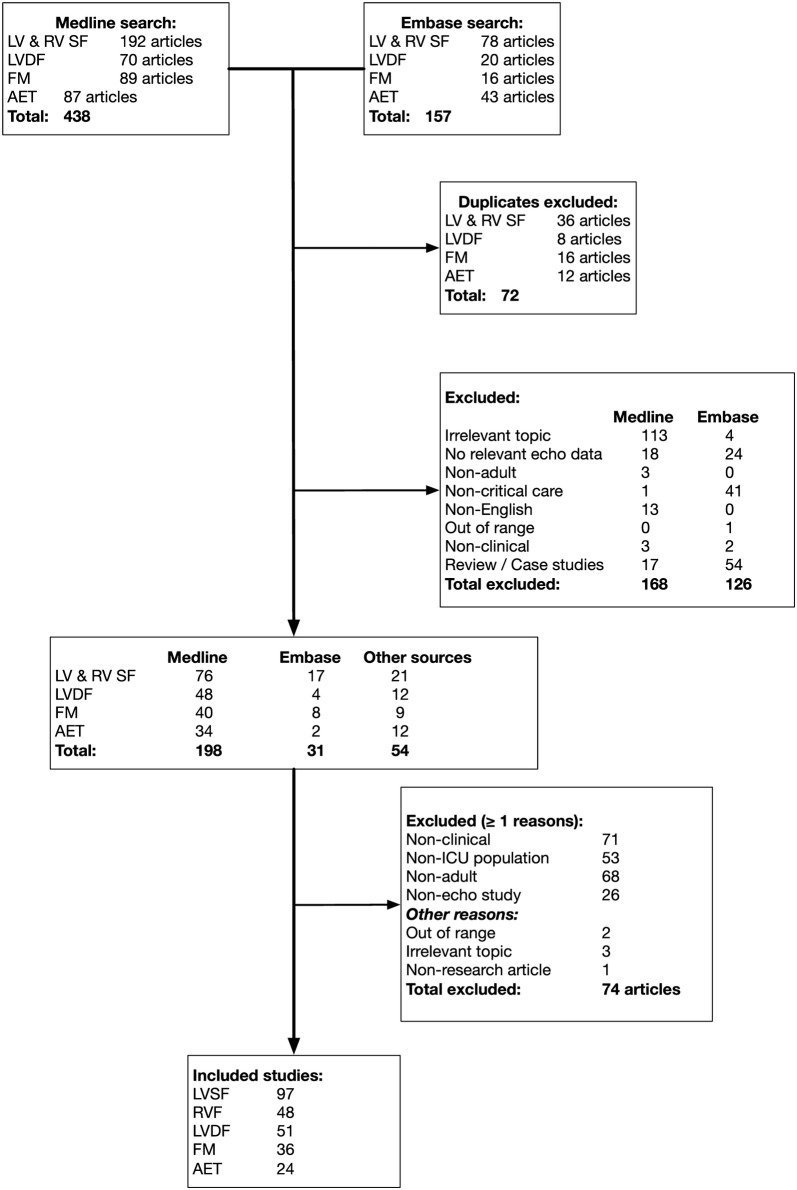
Flowchart of the literature search. *AET* advanced echocardiography techniques, *FM* fluid management, *LVDF* left ventricular diastolic function, *LVSF* left ventricular systolic function, *RVF* right ventricular function

**Fig. 2:**
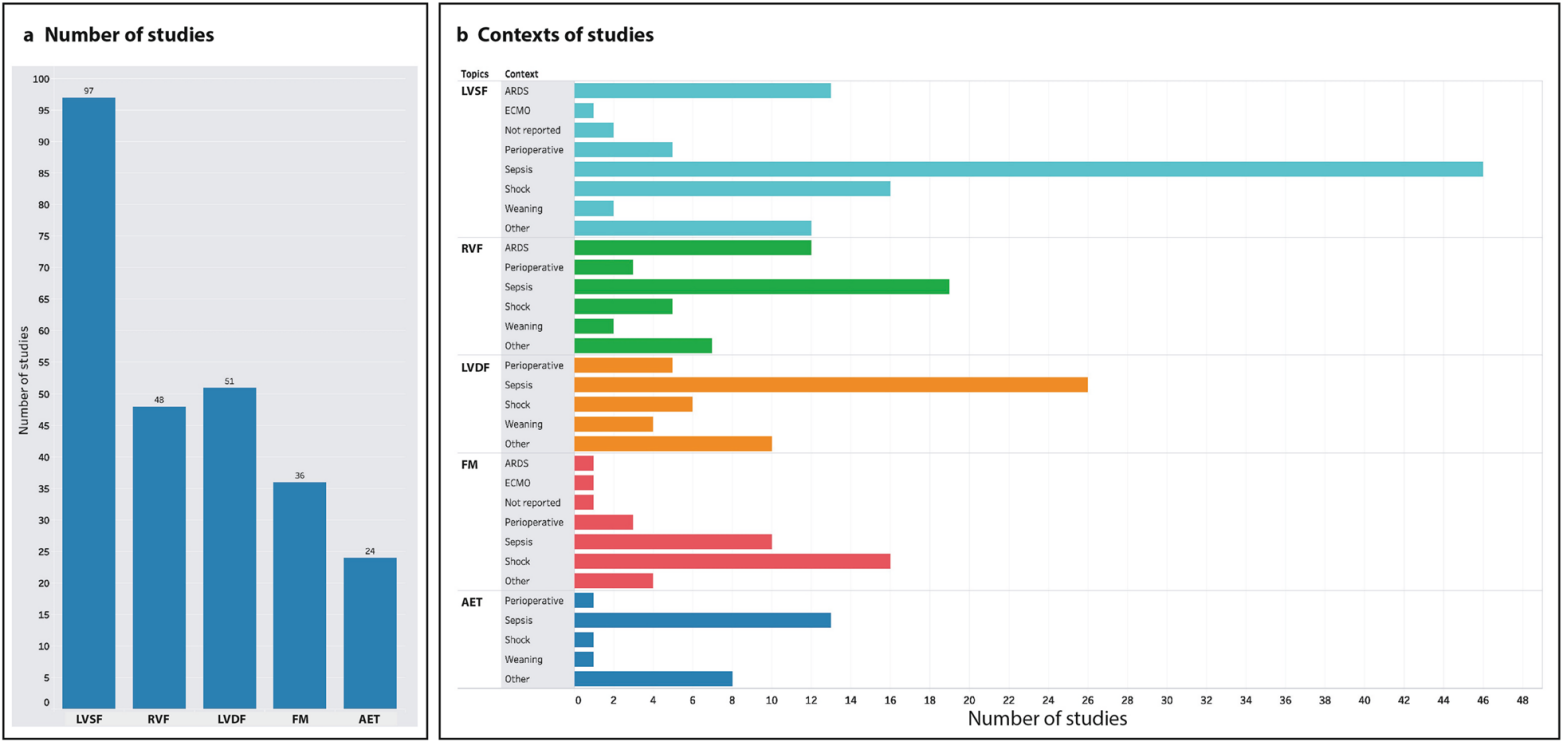
Number (**a**) and clinical context (**b**) of the included studies included into the systematic review, per topics. *AET* advanced techniques, *ARDS* acute respiratory distress syndrome, *ECMO* extracorporeal membrane oxygenation, *FM* fluid management, *LVDF* left ventricular diastolic function, *LVSF* left ventricular systolic function, *RVF* right ventricular function

Figure 1 shows the flow diagram for the literature search process. Medline and Embase returned 438 and 157 articles of which 72 were duplicates. After the exclusion of 294 articles based on abstract screening, 229 articles remained. Fifty-four articles were cross-referred during screening to other groups resulting in a total of 283 articles. The full-texts were appraised in detail, resulting in further exclusion of 74 articles. A total of 209 articles were finally included, some of which were assigned to more than one topic group (LVSF 97, RVF 48, LVDF 51, FM 36, and AET 24) (Fig. 2a).

### Summary of reporting of “common items” (43 items)

**Fig. 3:**
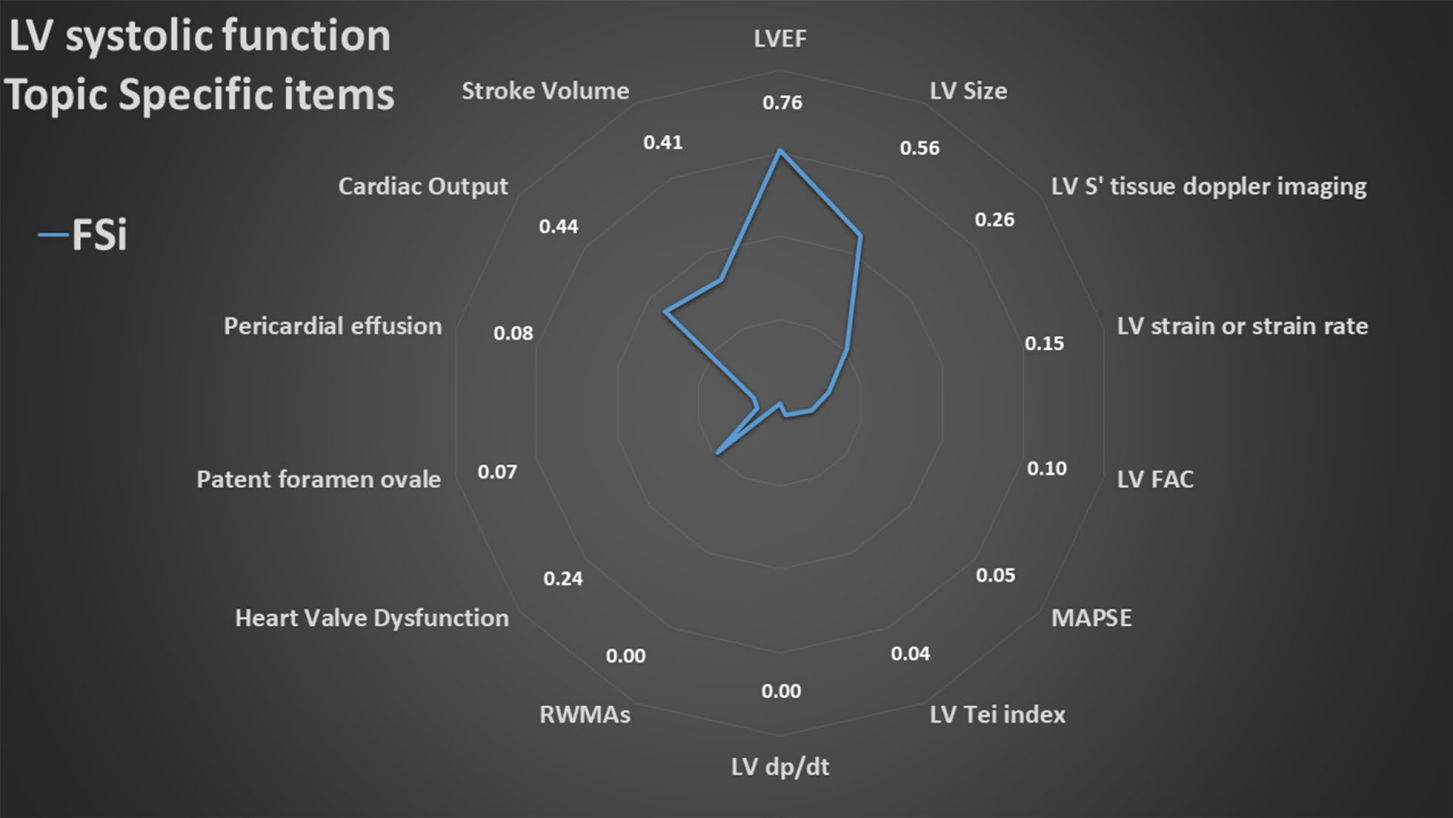
Radar plot of the fraction of studies reporting an item (FSi) in the left ventricular (LV) systolic function topic. *HFrEF* history of heart failure with reduced ejection fraction, *LVEF* LV ejection fraction, *LVFAC* LV fractional area change, *MAPSE* mitral annulus plan systolic excursion, *RWMAs* regional wall motion abnormalities, *Sʹ* maximal systolic velocity by tissue Doppler imaging at the mitral annulus. As example, an FSi score of 0.76 for LVEF means that 76% of studies on LV systolic function reported LVEF

**Fig. 4:**
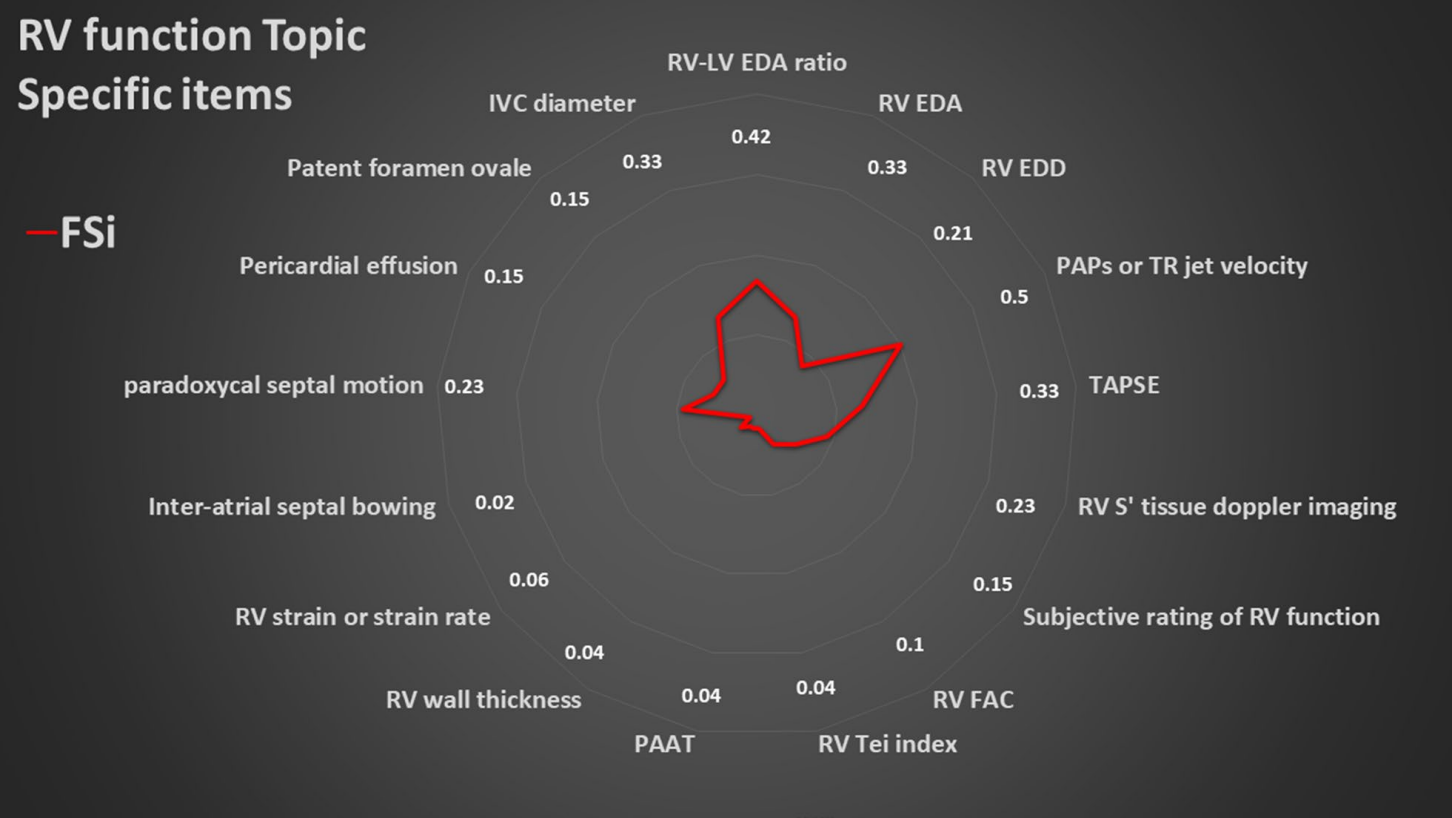
Radar plot of the fraction of studies reporting an item (FSi) in the right ventricular (RV) function topic. *IVC* inferior vena cava, *LV* left ventricle, *PAAT* pulmonary acceleration time, *PAPs* pulmonary artery systolic pressure, *RVEDA* RV end-diastolic area, *RVEDD* RV end-diastolic diameter, *RV FAC* RV fractional area change, *TAPSE* tricuspid annulus systolic excursion, *TR* tricuspid regurgitation, *Sʹ* maximal systolic velocity by tissue Doppler imaging at the tricuspid annulus. As example, an FSi score of 0.42 for RV-LV EDA ratio means that 42% of studies on RV function reported RV-LV EDA ratio

**Fig. 5:**
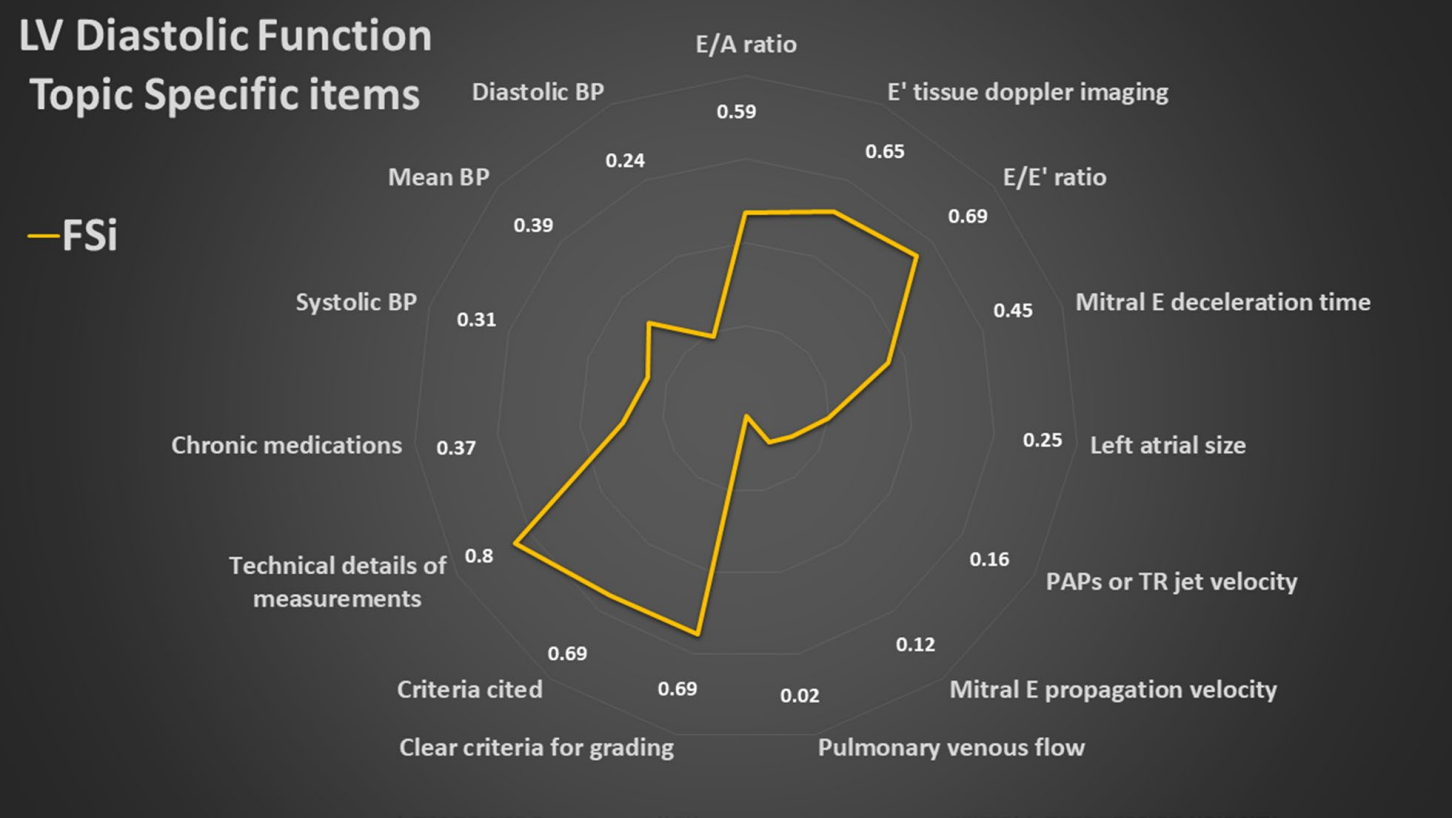
Radar plot of the fraction of studies reporting an item (FSi) in the left ventricular (LV) diastolic function topic. *A* atrial wave of transmitral diastolic blood flow, *BP* blood pressure, *E* early wave of transmitral diastolic blood flow, *Eʹ* maximal diastolic early velocity by tissue Doppler imaging at the mitral annulus, *PAPs* pulmonary artery systolic pressure, *TR* tricuspid regurgitation. As example, an FSi score of 0.59 for *E*/*A* ratio means that 59% of studies on LV diastolic function reported *E*/*A* ratio

**Fig. 6:**
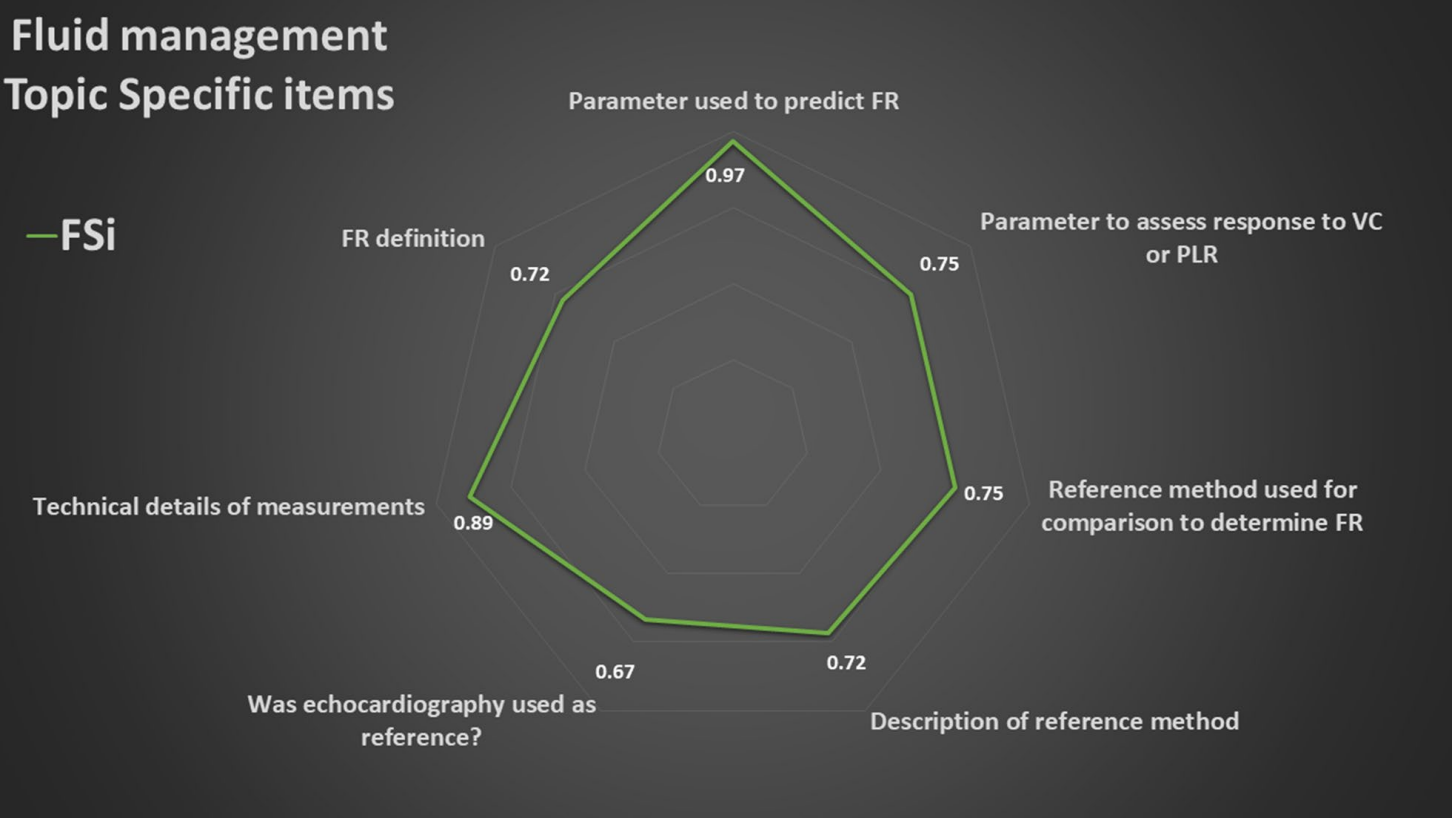
Radar plot of the fraction of studies reporting an item (FSi) in the fluid management topic. *FR* fluid responsiveness, *PLR* passive leg raising, *VC* volume challenge. As example, an FSi score of 0.72 for FR definition means that 72% of studies on fluid management reported FR definition

**Fig. 7:**
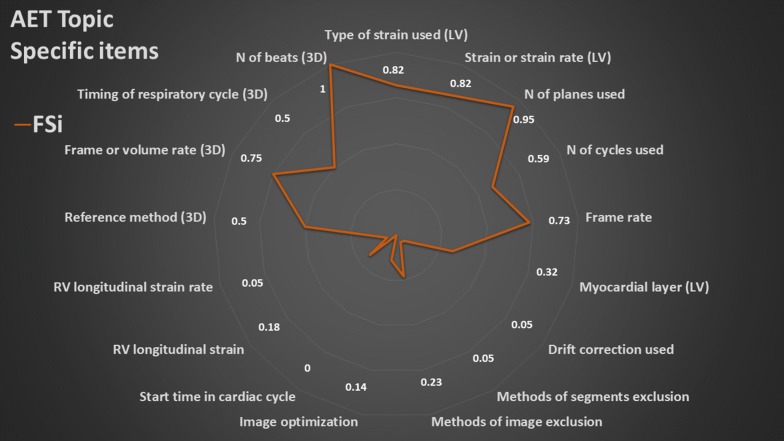
Radar plot of the fraction of studies reporting an item (FSi) in the advanced echocardiography techniques (AET) topic. All parameters but the last four in anticlockwise sense starting at 12 o’clock refers to strain echocardiography method. The last four refers to three-dimensional echocardiography (3-D) method. LV: left ventricle, RV: right ventricle. As example, an FSi score of 0.82 for type of strain used for LV studies means that 82% of studies reported the type of strain used to evaluate LV function

A total of 43 items common to all CCE topics were extracted. The values of FSi for each item are provided according to the topic of interest for the main ones (Figs. 3, 4, 5, 6, 7) and extensively as Additional files 2, 3, 4, 5, 6.

#### Study characteristics (3 items)

All studies reported the sample size. Most studies were prospective observational (87%), while interventional studies accounted for about 10% and the remaining were retrospective or post hoc studies.

#### Patients characteristics (12 items)

Clinical context, age and gender had high FSi. The clinical context varied among the CCE topics, with sepsis accounting for 40% to 54% in all topics except for FM where only 28% were sepsis-related and most were on shock (44%). Shock and acute respiratory distress syndrome were relatively common among LVSF articles (both around 15%), while acute respiratory distress syndrome comprised 25% of RVF articles (Fig. 2b). Age and gender were reported in over 90% of studies across all topics, but < 50% of studies reported height and weight, or body mass index. Among past medical history data, atrial fibrillation was mentioned in about 40% of studies, mostly as exclusion criteria. The rate of reporting for other patients comorbidities was relatively low (< 30%).

#### Echocardiography: information and purpose (6 items)

Transthoracic echocardiography was the main echocardiography mode: the highest was the AET topic (87%), followed by FM (81%), LVDF (78%), LVSF (70%) and RVF (63%). Only 10–20% of the studies used transesophageal echocardiography or both in each topic. Apart from FM studies, the reports of image acquisition information were sub-optimal (e.g. < 40% reporting whether or not images were collected at end-expiration, or number of cardiac cycles used for averaging).

#### Clinical information during echocardiography procedure (10 items)

On average, over 65% articles in each topic reported the heart rate and blood pressure, except for the FM topic where > 80% of articles reported these information. Cardiac rhythm was reported in almost 50% of the studies; the use of inotropes, vasopressors, and their doses were reported in 49%, 68% and 43%, respectively. Regarding mechanical ventilation, the mode was described by 75% of studies, while the ventilatory settings in the case of mechanical ventilation, namely positive end-expiratory pressure, plateau pressure and tidal volume, were rarely reported (32%, 19% and 28%, respectively). Even in FM group, only 50% to 60% of the studies reported this information. Most studies (> 90%) did not report if airway pressures were displayed on the ultrasound monitor.

#### Measurement reliability (8 items)

Approximately 30% and 45% of the studies did not report who performed and reviewed the echocardiography exams, respectively. In most cases, critical care physicians were responsible of both performing and reviewing the exams. The rate of cardiologist involved in performing echocardiography exams was 5% to 10% (LVSF, RVF and FM topic) and slightly higher for LVDF (16%) and AET topic (37%). The involvement of cardiologist in reviewing the exams were between 17 and 25%, except FM topic were it was sensibly lower (6%). Sonographers were also occasionally involved, but mainly in performing the studies only. The level of training of clinicians performing and reporting the exam was described in 41% and 25% of the articles, respectively. On average, 28% and 22% of the studies reported intra-observer and inter-observer variabilities, respectively; 33% reported the feasibility of echocardiography.

#### Statistics analysis (4 items)

Less than 25% of studies reported power and sample size calculation. The proportion of studies reporting if the statistical analyses were blinded varied grossly: 71% in AET, 43% in FM, 31% in LVDF, 27% both for LVSF and RVF. Adjustment for confounders followed a similar trend.

### Summary of topic-specific items

The overall results of the values of the FSi of each topic-specific item are presented in radar plots (Figs. 3, 4, 5, 6, 7). The greater the area of the plot itself, the better is the overall reporting for topic-specific items in studies regarding that topic.

#### LV systolic function (15 items, Fig. 3, Additional file 2)

The average PIPS for studies included in the LVSF topic was low (29.6%). LV ejection fraction was reported by 76% of studies, and in particular Simpson’s method, visual estimation or both were used in 54%, 20% and 2% of the studies, respectively; 24% of studies did not indicate their method for LV ejection fraction measurements. For studies reporting LV size (56%), LV end-diastolic diameter (23%), area (20%) and volume (28%) were used, with some reporting more than one parameter (12%). For studies reporting *S*ʹ wave at mitral annulus on tissue Doppler imaging (26%), 66% did not report the segments used, while the remaining reported medial (septal) (11%), lateral (14%) or average of the two walls (8%).

#### RV function (18 items, Fig. 4, Additional file 3)

The average PIPS was low for this topic (19.1%). For studies reporting RV dimensions, 42% used the RV-to-LV end-diastolic areas ratio, 33% the RV end-diastolic area, and 21% the RV end-diastolic diameter. 15% of studies used subjective ratings of RV function, and 17% did not report any parameter of function, except RV-to-LV end-diastolic areas ratio and paradoxical septal motion. Half of studies reported pulmonary artery systolic pressure (PAPs) directly or from tricuspid regurgitation jet velocity.

#### LV diastolic function (15 items, Fig. 5, Additional file 4)

The average PIPS was 42.8%. *E*/*E*ʹ, *E*ʹ wave at mitral annulus on tissue Doppler imaging and *E/A* were more commonly reported (67%, 63% and 58%, respectively) as compared with pulmonary artery pressure (PAPs, or surrogates) and left atrial size (15% and 25%, respectively). Regarding left atrial size, the parameter was reported as volume (14%), diameter (8%) and area (4%). PAPs measured directly was only reported in 4% of the studies, while 11% of studies used tricuspid regurgitation jet velocity as surrogate for PAPs. Technical details of measurements were mostly reported (80%). The criteria used for evaluating LVDF were quoted only in 69% of studies.

#### Fluid management (7 items, Fig. 6, Additional file 5)

The average PIPS was 78%. The methods used to assess fluid responsiveness was reported by nearly all studies (97%), and various methods were used (volume challenge 72%; variations of stroke volume or its surrogates 36%: change in inferior vena cava or superior vena cava, 33% and 8%, respectively; passive leg raising 17%). Over 90% of studies reported gave technical details of measurements, but definition of fluid responsiveness was not always clear (72%). Roughly three-quarters of studies reported if and which “gold” standard for comparison was adopted to define fluid responders.

#### Advanced echocardiographic technique (17 items, Fig. 7, Additional file 6)

The average PIPS was 42%. A total of 13 items were identified for speckle tracking studies and other four for the 3-D studies. Most of ventricular strain studies were performed on the LV (> 80%); strain was more used than strain rate. Global and longitudinal strains were the most commonly reported (42% and 46%, respectively). Only 13% and 8% of studies reported circumferential and radial strains, respectively. The type of LV strain used was not reported by 17% of studies. Acquisition and analysis information were reported with a different degree, from relatively high (frame rate 67%, number of planes used for global strain 88%) to rather low (use of drift correction and segment exclusion 4%, clear image optimization procedure 14%, no study reporting the start time of recording).

Regarding 3-D echocardiography, technical information were all seldomly reported.

## Discussion

This systematic review summarizes the research reporting practice in CCE for studies published between year 2000 and 2017. The aim of the systematic review was to inspect past studies in order to describe reporting attitude and to identify potential areas of weakness and insufficient reporting, finally providing a robust evidence base for the expert panel to design recommendations for standardized reporting of future studies. Our goal is not to judge the quality of the past studies, nor to create unreasonable standards that could limit in the future the publication of interesting studies unable to report all the necessary items. Of note, studies from authors of the PRICES panel were evaluated in the same manner in this systematic review, and we found many of them had the same weaknesses and insufficiencies in reporting as the other researchers.

Our systematic review identified a considerable heterogeneity between studies and between the different fields of interest. For instance, studies in FM topic reported items in a higher number while those on LVDF topic lacked many items. Several items were under-reported despite their importance from either a methodological or clinical perspective. A large volume of narrative information was collected during the course of this work, but the discussion of all these findings would make the manuscript unnecessarily long, so we chose to present a limited sample to illustrate the level of under-reporting of important items in CCE studies. For example—the presence of atrial fibrillation at the time of echocardiography was mentioned only in a minority of studies (mainly as exclusion criteria) while it is known that its incidence during critical illness is relatively high [11–13] and that it may induce cardiac dysfunction (especially diastolic) and it complicates or invalidates most echocardiographic measurements. Moreover, it precludes the use of AET which requires normal sinus rhythm. Another example, despite the frequent use of vasoactive drugs in intensive care which are known to affect the interpretation of most echocardiographic variables, the presence and dosage of inotropes and vasopressors were sub-optimally reported (49%, 68% and 43%, respectively). This would clearly introduce a source of bias when comparing studies. Furthermore, the mode of ventilation was described by three-quarters of studies; however, the values of positive end-expiratory pressure, plateau pressure and tidal volume during the echocardiography examination were only reported in a minority of cases despite ventilation settings are known to affect heart performance and especially the RV function. Additionally, these omissions will limit the validity of echocardiography parameters in the investigation of fluid responsiveness [14].

We also evaluated methodological aspects of echocardiography studies and data analysis in each study. Among others, it appears that assessment for confounders, blinding, identification of the person responsible of both performing and reviewing the echocardiography studies are far from being systematically reported. We also found under-reporting of the “topic-specific” items, where one ideally would expect higher reporting due to their specificity for the area of interest. For instance, the LV ejection fraction was the most commonly used parameter to describe LVSF (76%), but information on LV size were provided in roughly half of studies. Information on RV dimensions were under-reported to a similar extent and RV wall thickness was seldom reported, despite the role of these measurements in signalling the effect of chronic lung disease on the RV [15]. Surprisingly, in the investigation of LVDF we found that in around one-third of cases the authors did not refer to existing guidelines [16, 17] and used their own criteria or quoted references other than guidelines. Similarly, in the study of the fluid management over one-quarter of studies did not provide sufficient information about the reference (“gold”) standard method used to assess fluid responsiveness.

After reporting these examples, we would like to emphasize that the purpose of the present systematic review is to provide solid evidence for the expert panel to design recommendations for the reporting of studies utilizing CCE, rather than to criticize the quality of the body of research or to create unreasonable standards. The information on the frequency of reporting will be of course weighted against the importance of each item with the target of establishing the essential items that need mandatory reporting in CCE studies. The ultimate aim is to guide future CCE researchers to pursue a standardized approach in study design and reporting to enhance reproducibility and data homogeneity. This will increase the external validity and the impact of individual studies, facilitating meaningful comparison and the pooling of data in meta-analyses. Similar to the rationale for the “PRISMA statement” [18], which provides structured guidance on the information that authors should report in systematic review and meta-analysis to improve data consistency and allowing meaningful pooling of results, the next step of the PRICES project is to construct recommendations based on this systematic review balanced with expert opinion on the importance of the appraised items.

## Limitations

Our study has some limitations. First, in chronological terms, our appraisal was limited to studies published from 2000 until the end of 2017; although it is likely that more recent studies have higher reporting scores, it is also probable that articles published before 2000 had worse reporting scores. Therefore, we believe unlikely that FSi results would have changed significantly with the inclusion of more recent and older publications. Moreover, it must be noted that we decided not to investigate the evolution of the frequency of the reporting of the different items throughout the study period. We believe that changes over time in reporting certainly have happened for certain items. The items of the AET are one clear example due to the novelty of this echocardiography modality, but also LVDF seems another field where variations in reporting attitude have happened over the time due to appearance of new guidelines [17] where the use of some items has been reduced (i.e. deceleration time and pulmonary venous flow) while it increased for others (tissue Doppler imaging, left atrial size and tricuspid regurgitation jet) [18].

Second, one can say that some results were quite expected. In truth, we—as authors of CCE studies—were somewhat surprised of the sub-optimal reporting of items important for the interpretation of study findings. In other words, we expected better performance in reporting from ourselves. This further highlights the need for providing guidance in reporting CCE studies, even for people supposed to be experts in this field. It is interesting for the researchers to note that in many studies, the authors did not report parameters allowing accurate interpretation of study findings, such as the sub-optimal reporting of LV size in studies regarding LVSF. On the other side, the absence of reporting of certain parameters are not surprising and as example we cannot be surprised that dP/dt was rarely reported in studies on LVSF, although some intensivists suggested the usefulness of this parameter [19].

Third, because we decided to perform our analysis by area of interest rather than by clinical situations which were regarded as too numerous and diverse, we acknowledge that some items identified by the experts could be inappropriate or difficult in certain settings. The most obvious situation is probably the use of CCE in cardiac arrest where nothing else than a qualitative evaluation is allowed, though it must be noted that studies on cardiac arrest do not focus on the topics we selected for the appraisal.

## Conclusions

This systematic review critically appraised the reporting pattern in over 15 years of CCE literature, and represents the first step in PRICES, an ESICM endorsed project that will produce recommendations for the reporting of CCE studies. This analysis confirmed sub-optimal reporting of a number of items, which if omitted are likely to bias study interpretation and reproducibility of its results. Despite all its limitations, the systematic description of the reporting attitude in CCE studies will be helpful for the construction of PRICES recommendations.

## Supplementary information

**Additional file 1.** Search strategies.

**Additional file 2.** Summary of reporting of LVSF items.

**Additional file 3.** Summary of reporting of RVF items.

**Additional file 4.** Summary of reporting of LVDF items.

**Additional file 5.** Summary of reporting of FM items

**Additional file 6.** Summary of reporting of AET items.

## Data Availability

Yes.

## Abbreviations

AET: Advanced echocardiography technique
CCE: Critical care echocardiography
ESICM: European Society of Intensive Care Medicine
FM: Fluid management
FSi: Fraction of studies reporting an item
LVDF: Left ventricular diastolic function
LVSF: Left ventricular systolic function
PIPS: Percentage of items per study
PAPs: Pulmonary artery systolic pressure
RVF: Right ventricular function
PRICES: Preferred reporting items for critical care echocardiography studies
