# Supplementary material for: Systematic review and literature appraisal on methodology of conducting and reporting critical-care echocardiography studies: a report from the European Society of Intensive Care Medicine PRICES expert panel
Source: Ann Intensive Care. 2020 Apr 25;10:49. doi: 10.1186/s13613-020-00662-y (PMC7183522; doi:10.1186/s13613-020-00662-y)

# Additional file 1

## Search strategies for each topic

### Topics: LV systolic function and RV function

*Given that LV systolic function and RV function can be reported separately or together [i.e. LVSF ∪ RVF ∪ (LVSF ∩ RVF)], these two topics were combined in the search strategy, but were separated during the screening and appraisal process.*


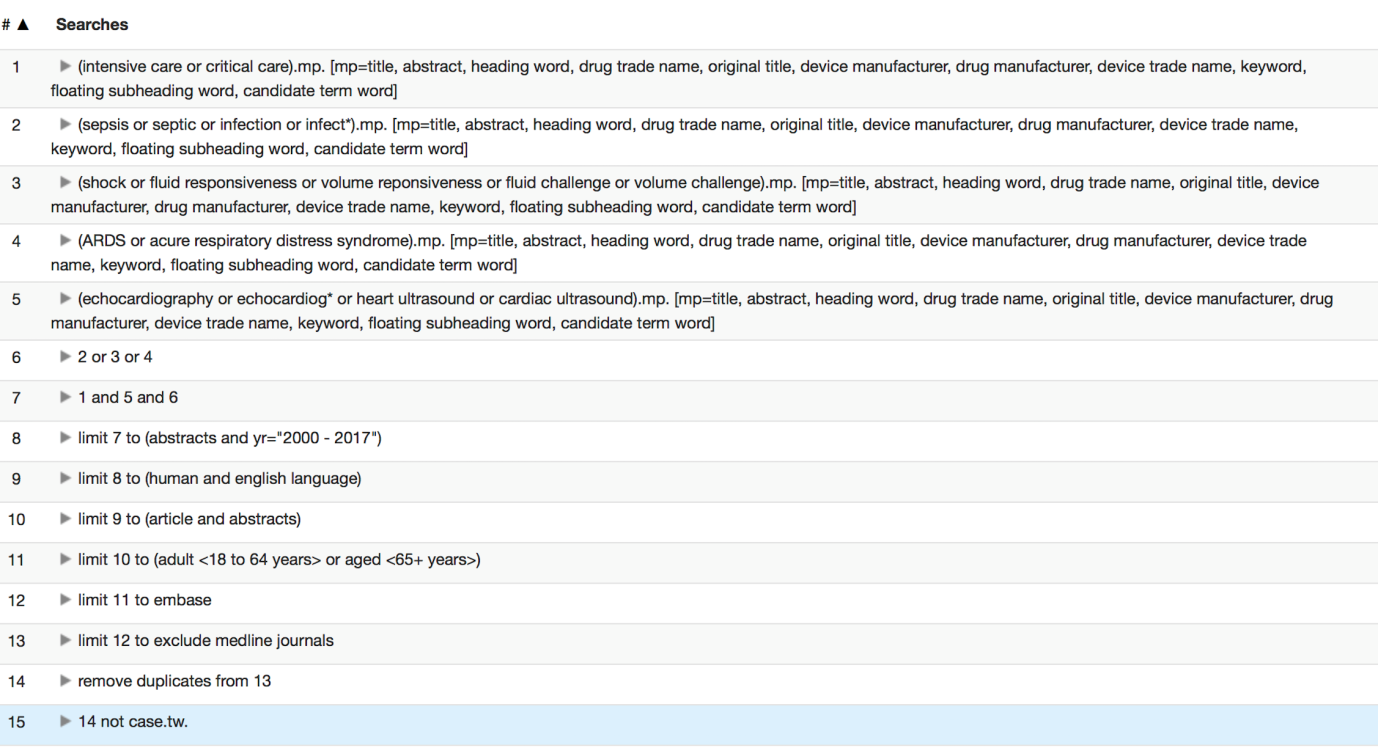


### Topics: LV diastolic function and fluid management


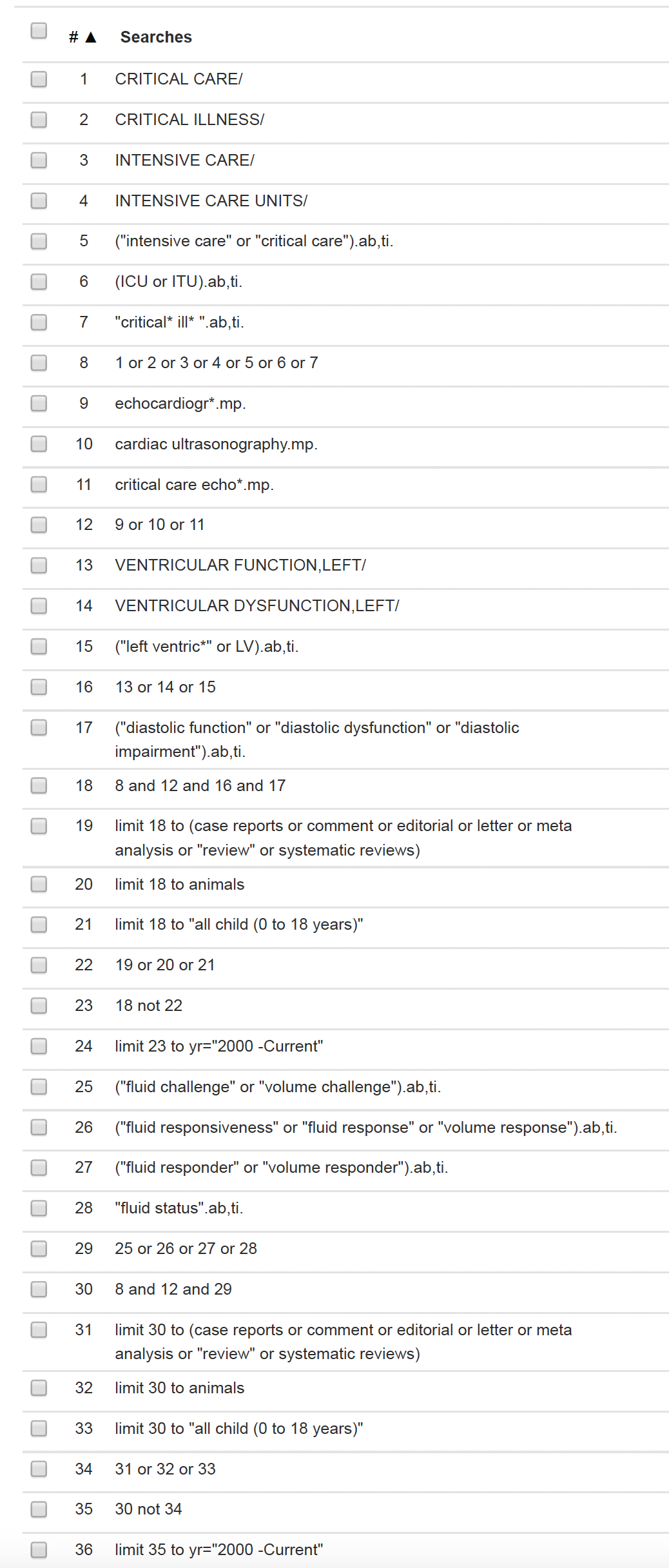


### Topic: Advanced echocardiography techniques


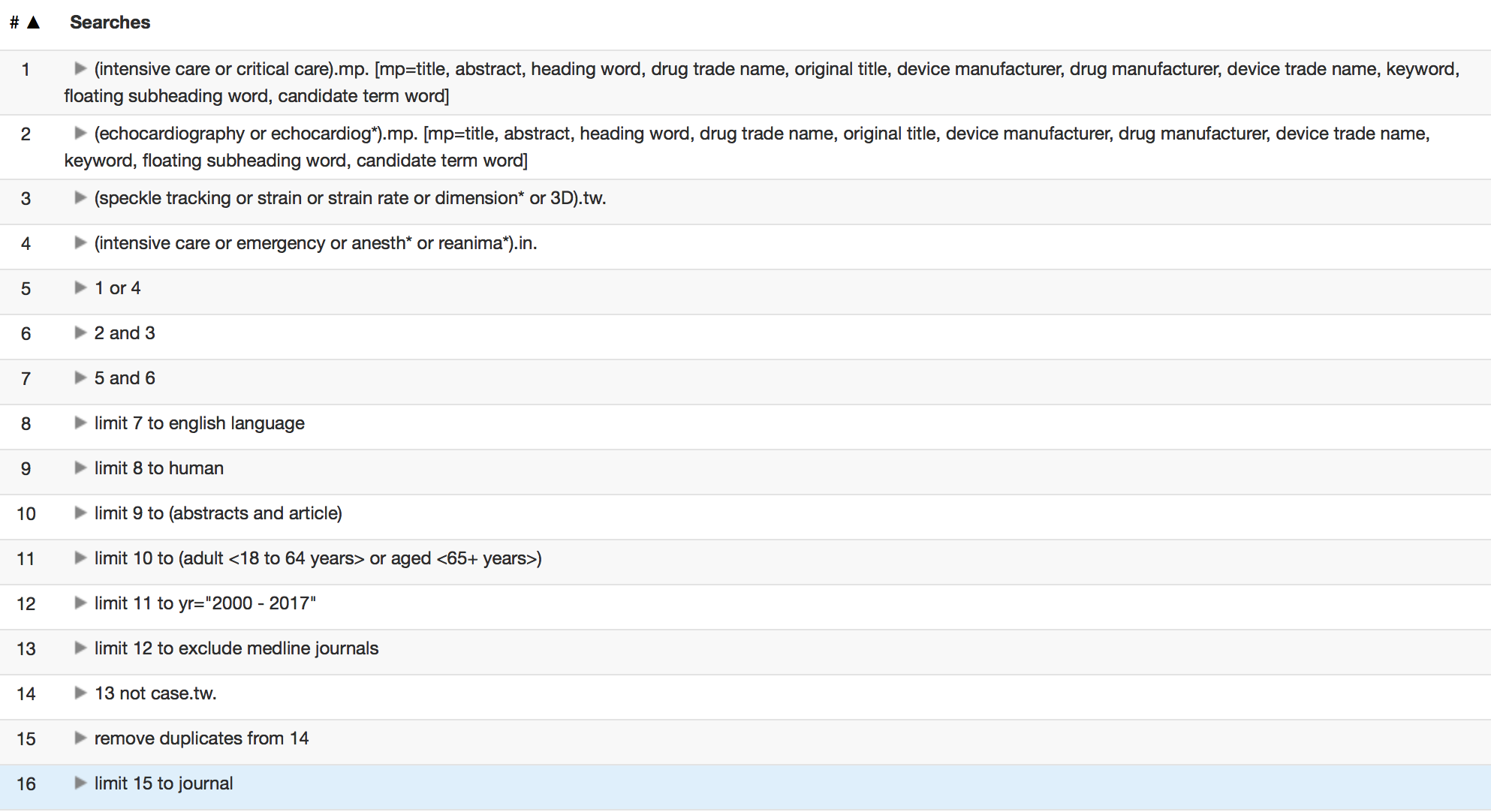

Supplement: Supplementary file 1 — Additional file 1. Search strategies. [file 13613_2020_662_MOESM1_ESM.docx]
