# Supplementary material for: Systematic review and literature appraisal on methodology of conducting and reporting critical-care echocardiography studies: a report from the European Society of Intensive Care Medicine PRICES expert panel
Source: Ann Intensive Care. 2020 Apr 25;10:49. doi: 10.1186/s13613-020-00662-y (PMC7183522; doi:10.1186/s13613-020-00662-y)
Supplement: Supplementary file 2 — Additional file 2. Summary of reporting of LVSF items. [file 13613_2020_662_MOESM2_ESM.docx]

# Additional file 2

**Fraction of studies (FSi)**

**of preferred items for left ventricular systolic function**

|  | **Domains and items** | ***FSi*** |
| --- | --- | --- |
| **Common to all topics** |  |  |
|  | ***Study information*** |  |
|  | *Sample size* | *1* |
|  | ***Patients characteristics*** |  |
|  | *Context* | *0.98* |
|  | *Age* | *0.98* |
|  | *Gender* | *0.89* |
|  | *Height & weight (or BMI)* | *0.41* |
|  | *History of hypertension* | *0.37* |
|  | *History of HFpEF* | *0.09* |
|  | *History of HFrEF* | *0.22* |
|  | *History of ischemic heart disease* | *0.40* |
|  | *History of atrial fibrillation* | *0.29* |
|  | *Presence of Pacemaker* | *0.05* |
|  | *History of COPD* | *0.21* |
|  | *History of chronic renal failure* | *0.28* |
|  | ***Echocardiography information*** |  |
|  | *Type of echocardiography* | *0.70* |
|  | *Data collected at end-expiration?* | *0.22* |
|  | *Data average over n beats?* | *0.34* |
|  | *Airway pressure trace displayed on screen?* | *0.04* |
|  | *Vendor of ultrasound machine* | *0.82* |
|  | *Software version* | *0.29* |
|  | ***Clinical information at the time of echocardiography*** |  |
|  | *Mode of ventilation* | *0.72* |
|  | *Tidal volume, if mechanically ventilated* | *0.25* |
|  | *Plateau pressure, if mechanically ventilated* | *0.20* |
|  | *PEEP, if mechanically ventilated* | *0.32* |
|  | *Cardiac rhythm* | *0.37* |
|  | *Heart rate* | *0.64* |
|  | *Blood pressure* | *0.60* |
|  | *Inotropes* | *0.53* |
|  | *Vasopressors* | *0.70* |
|  | *Doses of inotropes and vasopressors* | *0.45* |
|  | ***Measurement reliability*** |  |
|  | *Feasibility* | *0.21* |
|  | *Intra-observer variability* | *0.20* |
|  | *Inter-observer variability* | *0.14* |
|  | *Was observer blinded to treatment?* | *0.36* |
|  | *Echocardiographer professional training* | *0.70* |
|  | *Echocardiographer’s experience in echocardiography* | *0.42* |
|  | *Reviewer’s professional training* | *0.55* |
|  | *Reviewer’s experience in echocardiography* | *0.25* |
|  | ***Statistics reporting*** |  |
|  | *Sample size and power calculation provided?* | *0.05* |
|  | *Was the analysis blinded?* | *0.27* |
|  | *Were confounders addressed?* | *0.34* |
|  | *Was internal validation provided?* | *0.15* |
|  |  |  |
| **Topic-specific items** | ***LV systolic function*** |  |
|  | *Pericardial effusion* | *0.08* |
|  | *Tamponade* | *0.06* |
|  | *Patent foramen ovale* | *0.07* |
|  | *LV size* | *0.56* |
|  | *LV ejection fraction* | *0.76* |
|  | *Tissue Doppler S’ velocity* | *0.26* |
|  | *MAPSE* | *0.05* |
|  | *LV dP/dt* | *0.00* |
|  | *LV Tei index* | *0.04* |
|  | *LV strain or strain rate* | *0.15* |
|  | *Regional wall motion score* | *0.00* |
|  | *LV fractional area change* | *0.10* |
|  | *Cardiac output* | *0.44* |
|  | *Stroke volume* | *0.41* |
|  | *Valvular disease or dysfunction* | *0.24* |

BMI; body mass index, COPD: chronic obstructive pulmonary disease, HRrEF: heart failure with reduced ejection fraction, HFpEF: heart failure with preserved ejection fraction; LV: left ventricle, MAPSE: mitral annular plan systolic excursion, PEEP: positive end-expiratory pressure.
