# Supplementary material for: Systematic review and literature appraisal on methodology of conducting and reporting critical-care echocardiography studies: a report from the European Society of Intensive Care Medicine PRICES expert panel
Source: Ann Intensive Care. 2020 Apr 25;10:49. doi: 10.1186/s13613-020-00662-y (PMC7183522; doi:10.1186/s13613-020-00662-y)
Supplement: Supplementary file 3 — Additional file 3. Summary of reporting of RVF items. [file 13613_2020_662_MOESM3_ESM.docx]

# Additional file 3

**Fraction of studies (FSi)**

**of preferred items for right ventricular function**

|  | **Domains and items** | ***FSi*** |
| --- | --- | --- |
| **Common to all topics** |  |  |
|  | ***Study information*** |  |
|  | *Sample size* | *1* |
|  | ***Patients characteristics*** |  |
|  | *Context* | *1* |
|  | *Age* | *0.96* |
|  | *Gender* | *0.90* |
|  | *Height & weight (or BMI)* | *0.40* |
|  | *History of hypertension* | *0.40* |
|  | *History of HFpEF* | *0.10* |
|  | *History of HFrEF* | *0.27* |
|  | *History of ischemic heart disease* | *0.46* |
|  | *History of atrial fibrillation* | *0.33* |
|  | *Presence of Pacemaker* | *0.06* |
|  | *History of COPD* | *0.31* |
|  | *History of chronic renal failure* | *0.40* |
|  | ***Echocardiography information*** |  |
|  | *Type of echocardiography* | *0.63* |
|  | *Data collected at end-expiration?* | *0.25* |
|  | *Data average over n beats?* | *0.31* |
|  | *Airway pressure trace displayed on screen?* | *0.06* |
|  | *Vendor of ultrasound machine* | *0.83* |
|  | *Software version* | *0.23* |
|  | ***Clinical information at the time of echocardiography*** |  |
|  | *Mode of ventilation* | *0.88* |
|  | *Tidal volume, if mechanically ventilated* | *0.33* |
|  | *Plateau pressure, if mechanically ventilated* | *0.35* |
|  | *PEEP, if mechanically ventilated* | *0.46* |
|  | *Cardiac rhythm* | *0.42* |
|  | *Heart rate* | *0.69* |
|  | *Blood pressure* | *0.65* |
|  | *Inotropes* | *0.60* |
|  | *Vasopressors* | *0.77* |
|  | *Doses of inotropes and vasopressors* | *0.38* |
|  | ***Measurement reliability*** |  |
|  | *Feasibility* | *0.21* |
|  | *Intra-observer variability* | *0.15* |
|  | *Inter-observer variability* | *0.04* |
|  | *Observer blinded to treatment* | *0.38* |
|  | *Echocardiographer professional training* | *0.77* |
|  | *Echocardiographer’s experience in echocardiography* | *0.50* |
|  | *Reviewer’s professional training* | *0.60* |
|  | *Reviewer’s experience in echocardiography* | *0.35* |
|  | ***Statistics reporting*** |  |
|  | *Sample size and power calculation provided?* | *0.00* |
|  | *Was analysis blinded?* | *0.27* |
|  | *Were confounders addressed?* | *0.33* |
|  | *Was internal validation provided?* | *0.10* |
|  |  |  |
| **Topic-specific items** | ***RV function*** |  |
|  | *Pericardial effusion* | *0.15* |
|  | *Tamponade* | *0.10* |
|  | *Patent foramen ovale* | *0.15* |
|  | *RV wall thickness* | *0.04* |
|  | *RV end-diastolic diameter* | *0.21* |
|  | *RV end-diastolic area* | *0.33* |
|  | *RV to LV end-diastolic area ratio* | *0.42* |
|  | *TAPSE* | *0.33* |
|  | *RV Fractional Area Change* | *0.10* |
|  | *Tissue Doppler S’ velocity* | *0.23* |
|  | *RV Tei index* | *0.04* |
|  | *RV strain or strain rate* | *0.06* |
|  | *Subjective rating of RV function* | *0.15* |
|  | *PAPs or TR peak velocity* | *0.50* |
|  | *PAAT* | *0.04* |
|  | *Paradoxical septal motion* | *0.23* |
|  | *IVC diameter* | *0.33* |
|  | *IAS bowing* | *0.02* |

COPD: chronic obstructive pulmonary disease, HRrEF: heart failure with reduced ejection fraction, HFpEF: heart failure with preserved ejection fraction, FAC: fractional area contraction, IAS: inter atrial septum, IVC: inferior vena cava, LV: left ventricle, RV: right ventricle, TAPSE: tricuspid annular plan systolic excursion, PAPs: pulmonary artery systolic pressure, TR: tricuspid regurgitation, PAAT: pulmonary artery acceleration time.
