# Supplementary material for: Systematic review and literature appraisal on methodology of conducting and reporting critical-care echocardiography studies: a report from the European Society of Intensive Care Medicine PRICES expert panel
Source: Ann Intensive Care. 2020 Apr 25;10:49. doi: 10.1186/s13613-020-00662-y (PMC7183522; doi:10.1186/s13613-020-00662-y)
Supplement: Supplementary file 4 — Additional file 4. Summary of reporting of LVDF items. [file 13613_2020_662_MOESM4_ESM.docx]

# Additional file 4

**Fraction of studies (FSi)**

**of preferred items for left ventricular diastolic function**

|  | **Domains and items** | ***FSi*** |
| --- | --- | --- |
| **Common to all topics** |  |  |
|  | ***Study information*** |  |
|  | *Sample size* | *1.00* |
|  | ***Patients characteristics*** |  |
|  | *Context* | *1.00* |
|  | *Age* | *1.00* |
|  | *Gender* | *0.96* |
|  | *Height & weight (or BMI)* | *0.41* |
|  | *History of hypertension* | *0.57* |
|  | *History of HFpEF* | *0.14* |
|  | *History of HFrEF* | *0.33* |
|  | *History of ischemic heart disease* | *0.49* |
|  | *History of atrial fibrillation* | *0.43* |
|  | *Presence of pacemaker* | *0.08* |
|  | *History of COPD* | *0.22* |
|  | *History of chronic renal failure* | *0.39* |
|  | ***Echocardiography information*** |  |
|  | *Type of echocardiography* | *0.78* |
|  | *Data collected at end-expiration?* | *0.25* |
|  | *Data average over n beats?* | *0.59* |
|  | *Airway pressure trace displayed on screen?* | *0.04* |
|  | *Vendor of ultrasound machine* | *0.88* |
|  | *Software version* | *0.22* |
|  | ***Clinical information at the time of echocardiography*** |  |
|  | *Mode of ventilation* | *0.71* |
|  | *Tidal volume, if mechanically ventilated* | *0.10* |
|  | *Plateau pressure, if mechanically ventilated* | *0.02* |
|  | *PEEP, if mechanically ventilated* | *0.16* |
|  | *Cardiac rhythm* | *0.45* |
|  | *Heart rate* | *0.59* |
|  | *Blood pressure* | *0.51* |
|  | *Inotropes* | *0.45* |
|  | *Vasopressors* | *0.63* |
|  | *Doses of inotropes and vasopressors* | *0.45* |
|  | ***Measurement reliability*** |  |
|  | *Feasibility* | *0.43* |
|  | *Intra-observer variability* | *0.27* |
|  | *Inter-observer variability* | *0.29* |
|  | *Observer blinded to treatment* | *0.55* |
|  | *Echocardiographer professional training* | *0.69* |
|  | *Echocardiographer’s experience in echocardiography* | *0.37* |
|  | *Reviewer’s professional training* | *0.51* |
|  | *Reviewer’s experience in echocardiography* | *0.20* |
|  | ***Statistics reporting*** |  |
|  | *Sample size and power calculation provided?* | *0.14* |
|  | *Analysis was blinded?* | *0.43* |
|  | *Confounders addressed?* | *0.53* |
|  | *Internal validation provided?* | *0.00* |
|  |  |  |
| **Topic-specific items** | ***LV diastolic function*** |  |
|  | *Systolic blood pressure* | 0.31 |
|  | *Diastolic blood pressure* | 0.24 |
|  | *Mean arterial pressure* | 0.39 |
|  | *Chronic medications* | 0.37 |
|  | *E/A ratio* | 0.59 |
|  | *E’ velocity* | 0.65 |
|  | *E/E’ ratio* | 0.69 |
|  | *PAPs or Tricuspid regurgitation peak velocity* | 0.16 |
|  | *Left atrial size* | 0.25 |
|  | *Mitral E propagation velocity* | 0.12 |
|  | *Mitral E deceleration time* | 0.45 |
|  | *Pulmonary venous flow* | 0.02 |
|  | *Criteria used for grading diastolic function* | 0.69 |
|  | *Guidelines or reference for criteria cited* | 0.69 |
|  | *Technical details of measurements* | 0.80 |

COPD: chronic obstructive pulmonary disease, HRrEF: heart failure with reduced ejection fraction, HFpEF: heart failure with preserved ejection fraction, PAPs: pulmonary artery systolic pressure.
