# Supplementary material for: Systematic review and literature appraisal on methodology of conducting and reporting critical-care echocardiography studies: a report from the European Society of Intensive Care Medicine PRICES expert panel
Source: Ann Intensive Care. 2020 Apr 25;10:49. doi: 10.1186/s13613-020-00662-y (PMC7183522; doi:10.1186/s13613-020-00662-y)
Supplement: Supplementary file 5 — Additional file 5. Summary of reporting of FM items [file 13613_2020_662_MOESM5_ESM.docx]

# Additional file 5

**Fraction of studies (FSi)**

**of preferred items for fluid management**

|  | **Domains and items** | ***FSi*** |
| --- | --- | --- |
| **Common to all topics** |  |  |
|  | ***Study information*** |  |
|  | *Sample size* | *1.00* |
|  | ***Patients characteristics*** |  |
|  | *Context* | *0.94* |
|  | *Age* | *0.94* |
|  | *Gender* | *0.47* |
|  | *Height & weight (or BMI)* | *0.11* |
|  | *History of hypertension* | *0.08* |
|  | *History of HFpEF* | *0.03* |
|  | *History of HFrEF* | *0.06* |
|  | *History of ischemic heart disease* | *0.39* |
|  | *History of atrial fibrillation* | *0.00* |
|  | *Presence of pacemaker* | *0.14* |
|  | *History of COPD* | *0.03* |
|  | *History of chronic renal failure* | *0.94* |
|  | ***Echocardiography information*** |  |
|  | *Type of echocardiography* | *0.81* |
|  | *Data collected at end-expiration?* | *0.50* |
|  | *Data average over n beats?* | *0.56* |
|  | *Airway pressure trace displayed on screen?* | *0.14* |
|  | *Vendor of ultrasound machine* | *0.83* |
|  | *Software version* | *0.00* |
|  | ***Clinical information at the time of echocardiography*** |  |
|  | *Mode of ventilation* | *0.75* |
|  | *Tidal volume, if mechanically ventilated* | *0.61* |
|  | *Plateau pressure, if mechanically ventilated* | *0.33* |
|  | *PEEP, if mechanically ventilated* | *0.56* |
|  | *Cardiac rhythm* | *0.58* |
|  | *Heart rate* | *0.83* |
|  | *Blood pressure* | *0.83* |
|  | *Inotropes* | *0.44* |
|  | *Vasopressors* | *0.69* |
|  | *Doses of inotropes and vasopressors* | *0.47* |
|  | ***Measurement reliability*** |  |
|  | *Feasibility* | *0.33* |
|  | *Intra-observer variability* | *0.47* |
|  | *Inter-observer variability* | *0.42* |
|  | *Observer blinded to treatment* | *0.36* |
|  | *Echocardiographer professional training* | *0.72* |
|  | *Echocardiographer’s experience in echocardiography* | *0.33* |
|  | *Reviewer’s professional training* | *0.50* |
|  | *Reviewer’s experience in echocardiography* | *0.14* |
|  | ***Statistics reporting*** |  |
|  | *Sample size and power calculation provided?* | *0.25* |
|  | *Was analysis blinded?* | *0.31* |
|  | *Were confounders addressed?* | *0.25* |
|  | *Was internal validation provided?* | *0.00* |
|  |  |  |
| **Topic-specific items** | ***Fluid management*** |  |
|  | *Parameter used to predict fluid responsiveness** | *0.97* |
|  | *Echocardiographic parameter to assess response to volume challenge or passive leg raising*** | *0.75* |
|  | *Reference (“gold”) standard used for comparison to determine fluid responders* | *0.75* |
|  | *Description of the reference standard**** | *0.72* |
|  | *Echocardiography used as reference standard?* | *0.67* |
|  | *Fluid responsiveness definition* | *0.72* |
|  | *Technical details of measurements* | *0.89* |

*examples are: (mini-)volume challenge, passive leg raising, respiratory variation of stroke volume (e.g., velocity-time integral of left ventricular outflow tract Doppler tracing), respiratory variation of left ventricular outflow tract Doppler maximal velocity, respiratory variation of superior or inferior vena cava, respiratory variation of pulse pressure.

**stroke volume or stroke distance (i.e., velocity-time integral of left ventricular outflow tract Doppler tracing), cardiac output or cardiac index.

***for instance (mini-)volume challenge, passive leg raising.

BMI: body mass index, COPD: chronic obstructive pulmonary disease, HRrEF: heart failure with reduced ejection fraction, HFpEF: heart failure with preserved ejection fraction.
