# Supplementary material for: Systematic review and literature appraisal on methodology of conducting and reporting critical-care echocardiography studies: a report from the European Society of Intensive Care Medicine PRICES expert panel
Source: Ann Intensive Care. 2020 Apr 25;10:49. doi: 10.1186/s13613-020-00662-y (PMC7183522; doi:10.1186/s13613-020-00662-y)
Supplement: Supplementary file 6 — Additional file 6. Summary of reporting of AET items. [file 13613_2020_662_MOESM6_ESM.docx]

# Additional file 6

**Fraction of studies (FSi)**

**of preferred items for advanced echocardiography technology**

|  | **Domains and items** | ***FSi*** |
| --- | --- | --- |
| **Common to all topics** |  |  |
|  | ***Study information*** |  |
|  | *Sample size* | *1.00* |
|  | ***Patients characteristics*** |  |
|  | *Context* | *1.00* |
|  | *Age* | *0.96* |
|  | *Gender* | *0.96* |
|  | *Height & weight (or BMI)* | *0.58* |
|  | *History of hypertension* | *0.42* |
|  | *History of HFpEF* | *0.04* |
|  | *History of HFrEF* | *0.13* |
|  | *History of ischemic heart disease* | *0.46* |
|  | *History of atrial fibrillation* | *0.46* |
|  | *Presence of pacemaker* | *0.17* |
|  | *History of COPD* | *0.21* |
|  | *History of chronic renal failure* | *0.17* |
|  | ***Echocardiography information*** |  |
|  | *Type of echocardiography* | *0.88* |
|  | *Data collected at end-expiration?* | *0.17* |
|  | *Data average over n beats?* | *0.54* |
|  | *Airway pressure trace displayed on screen?* | *0.00* |
|  | *Vendor of ultrasound machine* | *1.00* |
|  | *Software version* | *0.92* |
|  | ***Clinical information at the time of echocardiography*** |  |
|  | *Mode of ventilation* | *0.71* |
|  | *Tidal volume, if mechanically ventilated* | *0.13* |
|  | *Plateau pressure, if mechanically ventilated* | *0.04* |
|  | *PEEP, if mechanically ventilated* | *0.13* |
|  | *Cardiac rhythm* | *0.58* |
|  | *Heart rate* | *0.67* |
|  | *Blood pressure* | *0.71* |
|  | *Inotropes* | *0.42* |
|  | *Vasopressors* | *0.58* |
|  | *Doses of inotropes and vasopressors* | *0.38* |
|  | ***Measurement reliability*** |  |
|  | *Feasibility* | *0.83* |
|  | *Intra-observer variability* | *0.58* |
|  | *Inter-observer variability* | *0.42* |
|  | *Observer blinded to treatment* | *0.50* |
|  | *Echocardiographer professional training* | *0.67* |
|  | *Echocardiographer’s experience in echocardiography* | *0.46* |
|  | *Reviewer’s professional training* | *0.50* |
|  | *Reviewer’s experience in echocardiography* | *0.33* |
|  | ***Statistics reporting*** |  |
|  | *Sample size and power calculation provided?* | *0.21* |
|  | *Was analysis blinded?* | *0.71* |
|  | *Were confounders addressed?* | *0.58* |
|  | *Was internal validation provided?* | *0.13* |
|  |  |  |
| **Topic-specific items** | ***Advanced echocardiography techniques*** |  |
|  | *Types of strain used in LV study* | *0.82* |
|  | *Strain or strain rate used in LV study* | *0.82* |
|  | *RV longitudinal strain* | *0.18* |
|  | *RV longitudinal strain rate* | *0.05* |
|  | *Myocardial layer analysed for LV study* | *0.32* |
|  | *Number of cycles used in analysis* | *0.59* |
|  | *Start time in cardiac cycle used in analysis* | *0.00* |
|  | *Frame rate* | *0.73* |
|  | *Number of planes used in analysis* | *0.95* |
|  | *Method of image exclusion* | *0.23* |
|  | *Method of segments exclusion* | *0.05* |
|  | *Details of image optimization method* | *0.14* |
|  | *Drift correction used* | *0.05* |
|  | *Number of beats used in 3-D analysis* | *1.00* |
|  | *Frame or volume rate used in 3-D analysis* | *0.75* |
|  | *Timing of respiratory cycle in 3-D analysis* | *0.50* |
|  | *Reference method in 3-D analysis* | *0.50* |

COPD: chronic obstructive pulmonary disease, HRrEF: heart failure with reduced ejection fraction, HFpEF: heart failure with preserved ejection fraction, LV: left ventricle, RV: right ventricle.
